# Regulation of CYP3A genes by glucocorticoids in human lung cells

**DOI:** 10.12688/f1000research.2-173.v2

**Published:** 2013-10-08

**Authors:** Jessica K Roberts, Chad D Moore, Erin G Romero, Robert M Ward, Garold S Yost, Christopher A Reilly

**Affiliations:** 1 Department of Pharmacology and Toxicology, College of Pharmacy, University of Utah, Salt Lake City UT, 84112, USA; 2 Department of Pediatrics, School of Medicine, University of Utah, Salt Lake City UT, 84108, USA

## Abstract

Inhaled glucocorticoids are the first-line treatment for patients with persistent asthma.  However, approximately thirty percent of patients exhibit glucocorticoid insensitivity, which may involve excess metabolic clearance of the glucocorticoids by CYP3A enzymes in the lung.  CYP3A4, 3A5, and 3A7 enzymes metabolize glucocorticoids, which in turn induce CYP3A genes.  However, the mechanism of CYP3A5 mRNA regulation by glucocorticoids in lung cells has not been determined.  In hepatocytes, glucocorticoids bind to the glucocorticoid receptor (GR), which induces the expression of the constitutive androstane receptor or pregnane X receptor; both of which bind to the retinoid X receptor alpha, leading to the induction of CYP3A4, 3A5, and 3A7.  There is also evidence to suggest a direct induction of CYP3A5 by GR activation in liver cells. In this study, these pathways were evaluated as the mechanism for CYP3A5 mRNA induction by glucocorticoids in freshly isolated primary tracheal epithelial, adenocarcinomic human alveolar basal epithelial (A549), immortalized bronchial epithelial (BEAS-2B), primary normal human bronchial/tracheal epithelial (NHBE), primary small airway epithelial (SAEC), and primary lobar epithelial lung cells. In A549 cells, beclomethasone 17-monopropionate ([M1]) induced CYP3A5 mRNA through the glucocorticoid receptor. CYP3A5 mRNA induction by five different glucocorticoids was attenuated by inhibiting the glucocorticoid receptor using ketoconazole, and for beclomethasone dipropionate, using siRNA-mediated knock-down of the glucocorticoid receptor. The constitutive androstane receptor was not expressed in lung cells. SAEC cells, a primary lung cell line, expressed CYP3A5, but CYP3A5 mRNA was not induced by glucocorticoid treatment despite evaluating a multitude of cell culture conditions. None of the other lung cells expressed CYP3A4, 3A5 or 3A7 mRNA. These studies demonstrate that CYP3A5 mRNA is induced by glucocorticoids in A549 cells via the glucocorticoid receptor, but that additional undefined regulatory processes exist in primary lung cells.

## Introduction

**Figure 1.**
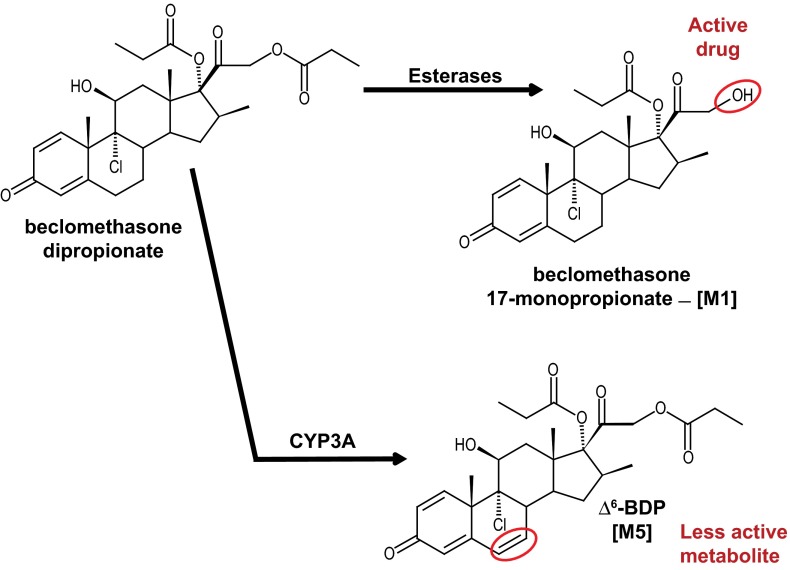
Metabolic scheme for the production of [M1] (the active form of the drug) by esterase enzymes and [M5] by CYP3A enzymes.

Inhaled glucocorticoids are the first-line treatment for asthma ^1– 3^. Glucocorticoids bind to the glucocorticoid receptor to reduce the expression of genes that produce a variety of pro-inflammatory mediators and mucus in the lung ^4– 6^. The most commonly prescribed glucocorticoids are beclomethasone dipropionate (BDP), triamcinolone acetonide (TCL), budesonide (BUD), fluticasone propionate (FLT), and flunisolide (FLN) ^1^. BDP is a pro-drug and requires removal of the C-21 propionate group to become pharmacologically active; the active drug is beclomethasone 17-monopropionate, referred to as [M1] ( Figure 1) ^7^. Pharmacological inactivation and clearance of glucocorticoids, such as BDP and its active metabolite [M1], is mediated, in part, by cytochrome P450 (CYP) enzymes ( Figure 1).

In humans, CYP3A4, 3A5, and 3A7 are the primary CYP enzymes involved in glucocorticoid metabolism ^8– 11^. CYP3A4 is the most abundant CYP3A enzyme in the liver and intestines ^8, 12, 13^, CYP3A5 is more prevalent in the lung than the liver ^12, 14– 16^, and CYP3A7 is expressed in fetal liver, but diminishes after birth when CYP3A4 becomes the dominant adult hepatic CYP3A enzyme ^17, 18^. Expression of CYP3A7 in fetal and adult respiratory tissue has also been observed ^16^.

**Figure 2.**
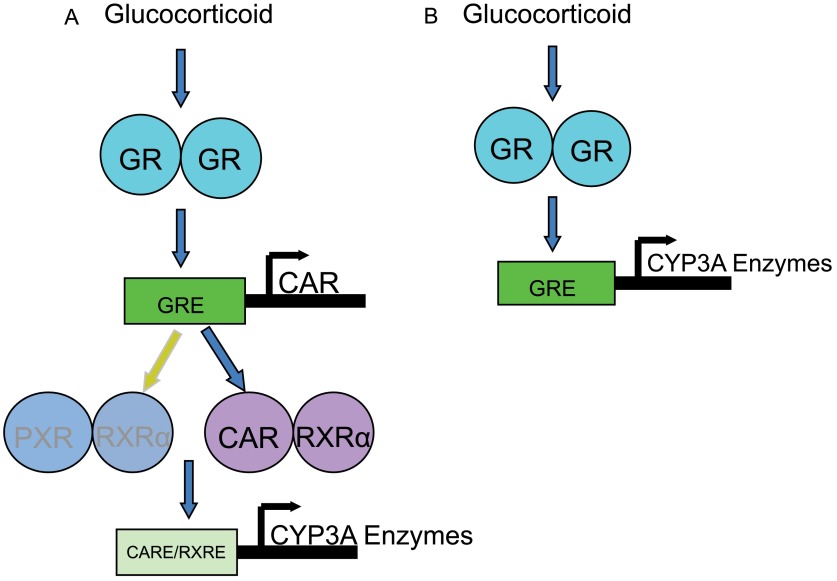
Possible mechanisms for the induction of CYP3A genes in lung cells. ( **A**) Active glucocorticoid will bind to the glucocorticoid receptor (GR), which forms a homodimer and translocates to the nucleus. The homodimer binds to its response element (GRE) and induces the expression of either the pregnane X receptor (PXR) or the constitutive androstane receptor (CAR). CAR or PXR (though this receptor is not expressed in the lungs) forms a heterodimer with the retinoic X receptor alpha (RXRα) which in turn induces the expression of the CYP3A enzymes via binding of the respective response-elements (CARE and/or PXRE). ( **B**) Active glucocorticoid will bind to the glucocorticoid receptor (GR), which forms a homodimer and translocates into the nucleus. The homodimer binds to its response element (GRE) and induces the expression of CYP3A enzymes.

Regulation of CYP3A enzymes in response to glucocorticoid treatment has been extensively characterized in the liver, but less is known about this phenomenon in the lung. In hepatocytes, CYP3A enzyme induction is mediated by the pregnane X receptor (PXR) ^19, 20^ ( Figure 2A). However, PXR is not expressed in the lung ^21^. Glucocorticoids can also influence CYP3A induction via the glucocorticoid receptor (GR) and the constitutive androstane receptor (CAR) in the liver ^22, 23^. Briefly, glucocorticoids bind GR in the cytosol, which forms a homodimer and translocates into the nucleus, leading to increased transcription of CAR. CAR forms a heterodimer with the retinoid X receptor alpha (RXRα), which binds to the RXR-response element and induces the expression of CYP3A enzymes ( Figure 2A) ^22^. Previous work by Hukannen *et al.* demonstrated that CAR was not expressed in A549 (adenocarcinomic human alveolar basal epithelial) cells and suggested that glucocorticoid binding to GR may directly regulate CYP3A gene expression in A549 cells ( Figure 2B), based on inhibition using RU-486 ^15, 24^. However, these pathways have not been evaluated in primary lung cell cultures or lung tissue.

The purpose of this study was three fold: to evaluate the changes in the expression of CYP3A mRNA in primary lung cells treated with glucocorticoids; to determine which pathway was responsible for glucocorticoid-induced changes in CYP3A mRNA expression; and to determine the role of BDP metabolism in this phenomenon. The cell lines used in this study were BEAS-2B (immortalized bronchial epithelial cell line), NHBE (normal human bronchial/tracheal epithelial cells), lobar epithelial cells (secondary bronchus epithelial cells), primary cells recovered from tracheal washes of pediatric patients on ventilation, SAEC (small airway epithelial cells), and A549 (human lung adenocarcinoma) cells. It was hypothesized that CYP3A5 mRNA induction in primary cells by BDP ^11^ and other glucocorticoids would occur via a mechanism involving GR/CAR/RXRα, as previously demonstrated using hepatocytes.

## Methods

### Chemicals, reagents, and treatments

Beclomethasone dipropionate (BDP), triamcinolone acetonide (TCL), fluticasone propionate (FLT), flunisolide (FLN), budesonide (BUD), prednisolone, ammonium acetate, eserine, and methanol were purchased from Sigma-Aldrich Chemical Company (St. Louis, MO). Paraoxon was purchased from Chem Service (West Chester, PA).

### Cell culture

A549 cells (American Type Culture Collection, Manassas, VA) were cultured in Dulbecco's Modified Eagle Medium (DMEM) fortified with 5% fetal bovine serum (Life Technologies, Grand Island, NY). SAEC cells (LONZA, Walkersville, MD; donor numbers 11662, 14453, 14457) were cultured in small airway epithelial growth medium, supplemented with the SAGM bullet kit (LONZA). Cells were cultured with and without hydrocortisone by adding or not adding the hydrocortisone component from the SAGM bullet kit. NHBE cells (LONZA; donor numbers 15268, 5S03795) were grown in bronchial epithelial cell growth medium (BEGM Bullet kit) (LONZA). BEAS-2B cells (American Type Culture Collection) were cultured in LHC-9 medium (Life Technologies). Lobar cells (donor number 01334) were cultured in BronchiaLife Basal Medium supplemented with the BronchiaLife B/T supplement kit (Lifeline Cell Technology, Walkersville, MD). All cells except A549 cells were plated in 12-well plates pre-coated with LHC basal medium (Life Technologies) and cultured in the presence of hydrocortisone. Tracheal epithelial cells were recovered from tracheal washes from mechanically ventilated pediatric patients in the neonatal intensive care unit and pediatric intensive care unit at Primary Children’s Medical Center at the University of Utah, with IRB approval (00026839). Briefly, cells were separated from sputum by centrifugation at 900 x g for 30 min in 14 mL of DMEM/F12 media. Cells were plated in a 12-well plate pre-coated with 2% gelatin (Life Technologies) and cultured in DMEM/F12 media + 10% fetal bovine serum (FBS) (Life Technologies). All cells were cultured in an atmosphere of 5% CO _2_:95% air at 37ºC.

### Cell treatments

Cell treatments were prepared in treatment media with a final concentration of DMSO less than 1%. Cells were treated at ~70% confluence. A549 cells were cultured in OPTIMEM (Life Technologies) and SAEC cells were cultured in growth media either with or without hydrocortisone and with or without heat inactivated and/or charcoal-stripped FBS. All other cell lines were treated in their respective growth medium, and heat inactivated to eliminate esterase activity from the FBS, which would metabolize BDP before it could diffuse into the cells. Cytotoxicity assays were performed using the Dojindo Cell Counting Kit-8 (Dojindo Laboratories, Rockville, MD) to determine glucocorticoid, esterase inhibitor, and ketoconazole concentrations exhibiting <20% cytotoxicity in A549 cells. All other cell lines were treated with the same concentrations as determined with A549 cells. Glucocorticoid treatments were as follows: BDP (10 μM), TCL (1 μM), BUD (10 μM), FLT (1 μM), and FLN (100 nM). Pre-treatments in various experiments included ketoconazole (Sigma-Aldrich Chemical Company) (50 μM, 10 μM, and 1 μM, to antagonize GR), esterase inhibitors (1:1 mixture of eserine and paraoxon, each at 175 μM, to inhibit [M1] formation), and 1-aminobenzotriazole (Sigma-Aldrich Chemical Company) (1-ABT; 200 μM, to inhibit P450-mediated metabolism) for 2 h prior to a 22 h glucocorticoid co-treatment. Controls were treated with an equivalent concentration of DMSO. All A549 cell treatments were carried out in 6-well plates for 24 h (n=6). All other cell lines were cultured in pre-coated 12-well plates and treated for 24 h (n=3).

### Analysis of BDP metabolites

After treatment, BDP and its metabolites were extracted from the collected media by adding 2x volume (6 mL for A549, 4 mL for all other cell lines) methyl *tert*-butyl ether containing 1 nM prednisolone (internal standard for quantification) and shaking for 25 min. Samples were clarified by centrifugation, the organic fraction was collected, dried under air, reconstituted in 100 µL 1:1 H _2_O:MeOH, clarified again by centrifugation, and transferred to autosampler vials for analysis by liquid chromatography-mass spectrometry (LC/MS/MS). LC/MS/MS was conducted on a Thermo LCQ Advantage Max ion trap instrument equipped with a Finnigan Surveyor LC pump, Surveyor Autosampler and universal Ion Max source operated with Thermo Xcalibur software version 2.0 (Thermo Fisher Scientific, Waltham, MA) as previously described ^11^.

### Quantitative reverse transcription-PCR

Total RNA was isolated from cells using TRIzol reagent (Life Technologies). cDNA was synthesized using iScript Reverse Transcription Supermix for qPCR (BIO RAD, Hercules, CA). qPCR was performed using either LightCycler 480 Probes Master mix (CYP3A5*1) or LightCycler 480 SYBR Green I Master Mix (all other genes) (Roche, Indianapolis, IN) with a LightCycler 480 System. The PCR program for the probe mix consisted of a 5 min incubation at 95ºC, followed by 45 cycles of 95ºC for 10s, 55ºC for 30s, then 72ºC for 1s. The PCR program for SYBR Green I mix consisted of a 5 min incubation at 95ºC, followed by 40 cycles of 95ºC for 10s, 63ºC for 5s for CYP3A4, CYP3A7 and β2-microglobulin. For GR and CAR, annealing was performed at 65ºC for 5s and extension at 72ºC for 10s. mRNA copy number was determined from standard curves for each gene and was normalized using β2-microglobulin. Primer sequences for the various genes are listed in Table 1 ^25^.

**Table 1.** Primer sequences for qPCR assays.

CYP3A5*1	F-5´ CCTATCGTCAGGGTCTCTGGAA 3´ R-5´ TGATGGCCAGCACAGGGA 3´ Probe [6FAM]ATGTGGGGAACGTATGAA[BHQ1]
CYP3A5-all	F-5´ CGTCAGGGTCTCTGGAAATTTG 3´ R-5´ CACGTCGGGATCTGTGATGG 3´
CYP3A4	F-5´ GAAAGTCGCCTCGAAGATAC 3´ R-5´ ACGAGCTCCAGATCGGACAG 3´
CYP3A7	F-5´ TTCCGTAAGGGCTATTGGAC 3´ R-5´ TCTGTGATAGCCAGCATAGG 3´
Glucocorticoid receptor	F-5´ CCAACGGTGGCAATGTGAAA 3´ R-5´ CCGCCAGAGGAGAAAGCAAA 3´
Constitutive androstane receptor	F-5´ CCGTGTGGGGTTCCAGGTAG 3´ R-5´ CAGCCAGCAGGCCTACGAAC 3´
β2- microglobulin	F-5´ GATGAGTATGCCTGCCGTGTG 3´ R-5´ CAATCCAAATGCGGCATCT 3´

### siRNA-mediated protein knockdown

Pre-annealed, short interfering “Smart Pool” siRNAs specific to human GR were purchased from Dharmacon (Waltham, MA). siRNA directed against GFP (negative control) ^26^ was synthesized at the University of Utah oligonucleotide synthesis core and annealed by combining 40 µM of each strand and incubating in annealing buffer (100 mM potassium acetate, 30 mM HEPES KOH, 2 mM magnesium acetate adjusted to pH 7.4) for 1 min at 90ºC followed by 1 h at 37ºC, in a final volume of 0.5 mL. A549 cells were plated into 6-well plates containing 20 nM siRNA per well, previously complexed with Lipofectamine 2000 (Life Technologies) using a ratio of 3:2 lipid to siRNA in 100 µL of OPTIMEM (Life Technologies). The cells were grown for 48, 72, and 96 h to determine the time at which maximum decreases in GR mRNA occurred (72 h). In subsequent experiments, cells were treated with DMSO, 10 µM BDP, or 10 μM BDP + 175 µM esterase inhibitors (1:1 eserine:paraoxon) for 24 h to determine the effects of attenuated GR expression on the induction of CYP3A5 in A549 cells.

### Statistical analysis

Statistical analysis was performed using GraphPad Prism 4.02 software for Windows (San Diego, CA). One-way ANOVA and Dunnett’s post-hoc test were used with p<0.05. All data are represented as a mean with error bars representing standard deviation.

## Results

### Inhibition of [M1] formation prevented CYP3A5 mRNA induction by BDP in A549 cells

**Figure 3.**
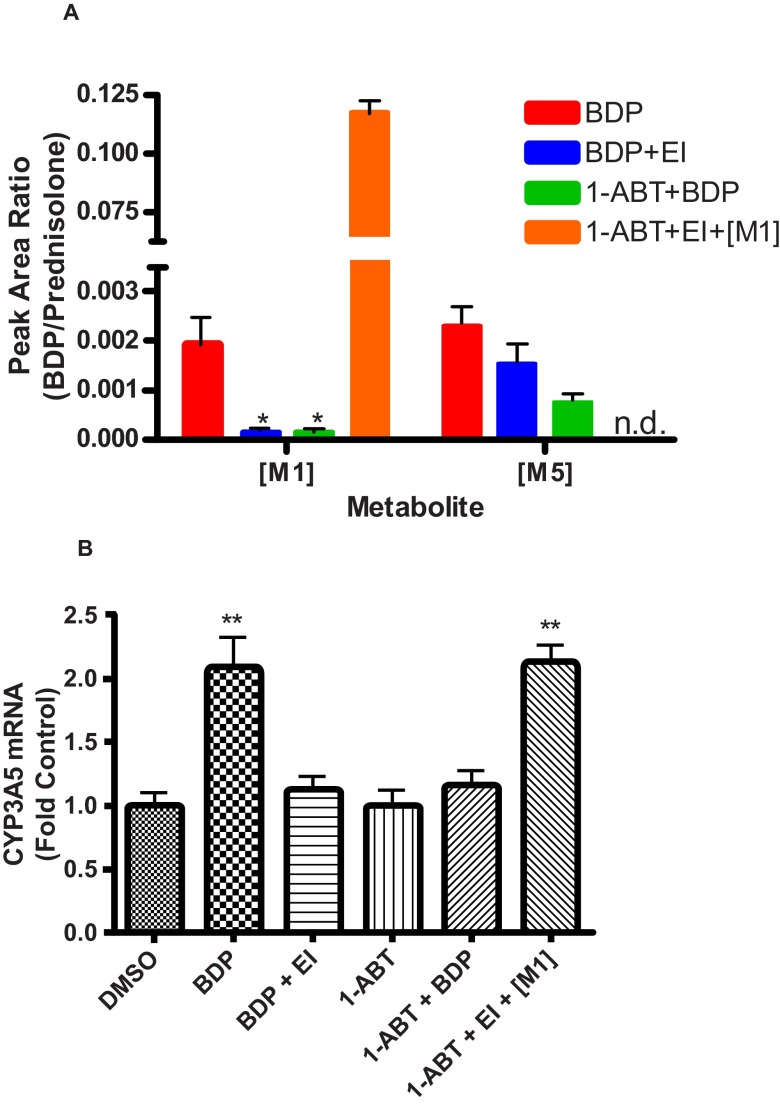
[M1] is required to induce CYP3A5 mRNA. ( **A**) Relative quantities of [M1] and [M5] measured by LC/MS/MS in A549 cell culture media following beclomethasone dipropionate (BDP) treatment alone, BDP + esterase inhibitors (EI), 1-aminobenzotriazole (1-ABT) + BDP, and 1-ABT + EI + [M1]. ( **B**) CYP3A5 mRNA detected in A549 cells following DMSO control, BDP treatment alone, BDP + EI, 1-ABT alone, 1-ABT + BDP, and 1-ABT + EI + [M1]. Values are expressed as fold over DMSO controls. Statistics used for data analysis were one-way ANOVA with Dunnett’s post-hoc test. Data are the mean and standard deviation from n=6 replicates. * p<0.05, ** p<0.01.

Media from A549 cells treated with BDP (10 µM) for 24 h was extracted and analyzed for metabolites of BDP produced by CYP3A enzymes. The only CYP3A-mediated metabolite detected was [M5] ( Figure 1 and Figure 3A) ^11^. For the remainder of the studies, [M1], the active metabolite, was used as a marker for esterase activity and [M5] was used as a marker for CYP3A5 activity. Only CYP3A5*1 mRNA was detected in A549 cells. CYP3A4 and CYP3A7 mRNA were not detected in A549 cells, as previously documented ^11, 16^. BDP treatment significantly induced the expression of CYP3A5 mRNA (~2-fold) compared to the DMSO control ( Figure 3B). Inhibiting the production of [M1] using esterase inhibitors also blocked the induction of CYP3A5 mRNA ( Figure 2A and Figure 2B); esterase inhibitor (EI) treatment alone had no effect on CYP3A5 expression. 1-ABT, a mechanism-based inactivator of P450 enzymes, also inhibited esterase activity (i.e. [M1] formation) ( Figure 3A), and as a result, prevented the induction of CYP3A5 mRNA ( Figure 3B). The mechanism by which 1-ABT inhibits esterases is not known.

### [M1] was sufficient to induce CYP3A5 mRNA in A549 cells

Cells were treated with [M1] in either the absence or presence of 1-ABT and esterase inhibitors. [M1] treatment was sufficient to induce CYP3A5 mRNA (~2-fold), without the requirement of esterases to produce [M1] ( Figure 3B), indicating that CYP3A5 mRNA induction in A549 cells was mediated by [M1].

### GR, but not CAR, regulated the induction of CYP3A5 mRNA in A549 cells

GR and CAR mRNA were quantified in A549 cells. A significant increase in GR mRNA (~2.5-fold) was observed following 24 h treatment with BDP ( Table 2), consistent with previous studies ^15^, suggesting that GR, not CAR, was responsible for the induction of CYP3A5 message in A549 cells.

**Table 2.** Comparison of glucocorticoid receptor (GR), constitutive androstane receptor (CAR), and CYP3A5 mRNA expression in lung cell cultures.

Cell type	GR expression	GR induction by GC treatment	CAR expression	CAR induction by GC treatment	CYP3A5 mRNA	CYP3A5 induction by GC treatment
Beas-2B	+	N.D.	N.D.	N.D.	N.D.	N.D.
NHBE	+	N.D.	N.D.	N.D.	N.D.	N.D.
Patient tracheal washes	N.D.	N.D.	N.D.	N.D.	N.D.	N.D.
Lobar	+	N.D.	N.D.	N.D.	N.D.	N.D.
A549	+	2.4 ± 0.35 **	N.D.	N.D.	+	2.1 ± 0.55 **
SAEC	+	N.D.	N.D.	N.D.	1 out of 3 patients	N.D.

Data are represented as fold over DMSO control. Statistics used for data analysis were one-way ANOVA with Dunnett’s post-hoc test. ** p<0.01, N.D. = not detected, GC = glucocorticoid.

### Inhibition of GR with ketoconazole attenuated CYP3A5 mRNA induction by glucocorticoids in A549 cells

**Figure 4.**
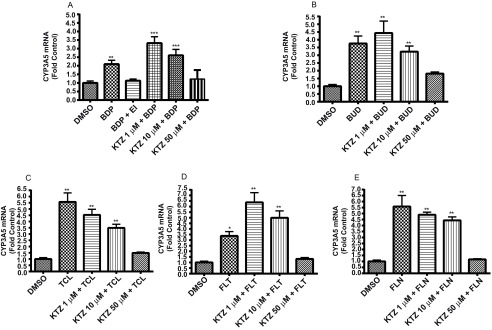
Ketoconazole inhibits the induction of CYP3A5 through the glucocorticoid receptor (GR). CYP3A5 mRNA detected in A549 cells treated with ( **A**) beclomethasone dipropionate (BDP), ( **B**) budesonide (BUD), ( **C**) triamcinolone acetonide (TCL), ( **D**) fluticasone propionate (FLT), and ( **E**) flunisolide (FLN), with and without ketoconazole (KTZ), a competitive antagonist for GR. Results are presented as fold over DMSO controls. Statistics used for data analysis were one-way ANOVA with Dunnett’s post-hoc test. Data are the mean and standard deviation from n=6 replicates. * p<0.05, ** p<0.01, *** p<0.001.

Ketoconazole is a competitive antagonist of GR ^25^. Ketoconazole alone had no significant effect on CYP3A5 mRNA expression as compared to DMSO controls. As the concentration of ketoconazole was decreased, dose-dependent increases in the expression of CYP3A5 mRNA were observed for BDP, TCL, FLT, BUD, and FLN ( Figure 4A–E): BDP caused a ~2-fold induction, BUD caused a ~4-fold induction, TCL caused a ~5.5-fold induction, FLT caused a ~3.5-fold induction, and FLN caused a ~5.5-fold induction, relative to their respective controls. These data confirm the hypothesis that the induction of CYP3A5 mRNA in A549 cells was mediated by GR. BDP or FLT paired with KTZ 1 µM treatment also showed further induction of CYP3A5 mRNA as compared to controls (~3.5 for BDP and ~6.5 for FLT). However, the basis and significance for this enhanced induction are not clear at this time.

### siRNA-mediated knockdown of GR also attenuated CYP3A5 mRNA induction by BDP in A549 cells

**Figure 5.**
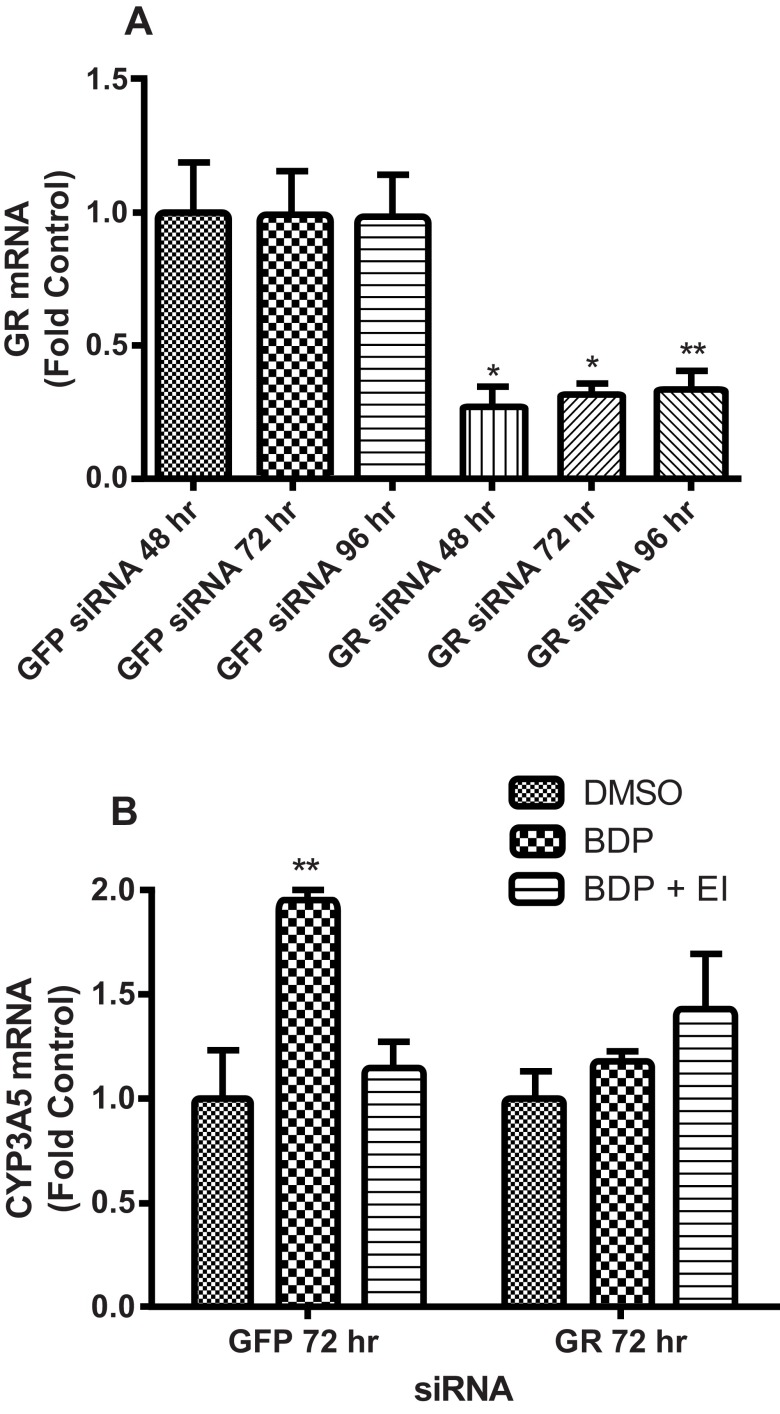
Glucocorticoid receptor (GR) siRNA blocks the induction of CYP3A5 by beclomethasone dipropionate (BDP). ( **A**) siRNA-mediated GR knockdown in A549 cells at 48, 72, 96 h compared to "nonsense" GFP siRNA (negative control), expressed as fold over designated GFP control for each time point. ( **B**) Cells were exposed to siRNA for 72 h then treated with DMSO, BDP, or BDP + esterase inhibitor (EI). Statistics used for data analysis were one-way ANOVA with Dunnett’s post-hoc test. Data are the mean and standard deviation from three replicates. * p<0.05, ** p<0.01.

Cells were transfected with siRNA and grown for 48, 72, and 96 h to determine the time of maximum GR mRNA knock down ( Figure 5A). Maximum suppression occurred as early as 48 h, but the 72 h time point was chosen for further experiments to ensure efficient GR protein depletion. An approximate 2-fold induction of CYP3A5 mRNA was observed in A549 cells following treatment with BDP in control cells transfected with “nonsense” siRNA directed against GFP. Consistent with previous results ( Figure 3A and Figure 3B), CYP3A5 mRNA induction was prevented by esterase inhibitors ( Figure 5B). Cells transfected with siRNA targeted for GR mRNA showed no change in CYP3A5 mRNA with BDP treatment ( Figure 5B), further confirming the role of GR in directly regulating the induction of CYP3A5 mRNA in A549 cells treated with BDP and presumably the other glucocorticoids used in Figure 4.

### CYP3A5 was not expressed or induced by glucocorticoid treatment in tracheal/bronchial epithelial cells

Neither CYP3A5*1 mRNA nor any other variant form of CYP3A5 mRNA was detected or induced by glucocorticoids in NHBE, BEAS-2B, lobar, and freshly isolated tracheal wash samples ( Table 2).

### SAEC cells expressed CYP3A5, but mRNA for CYP3A5 was not induced by glucocorticoid treatment

**Figure 6.**
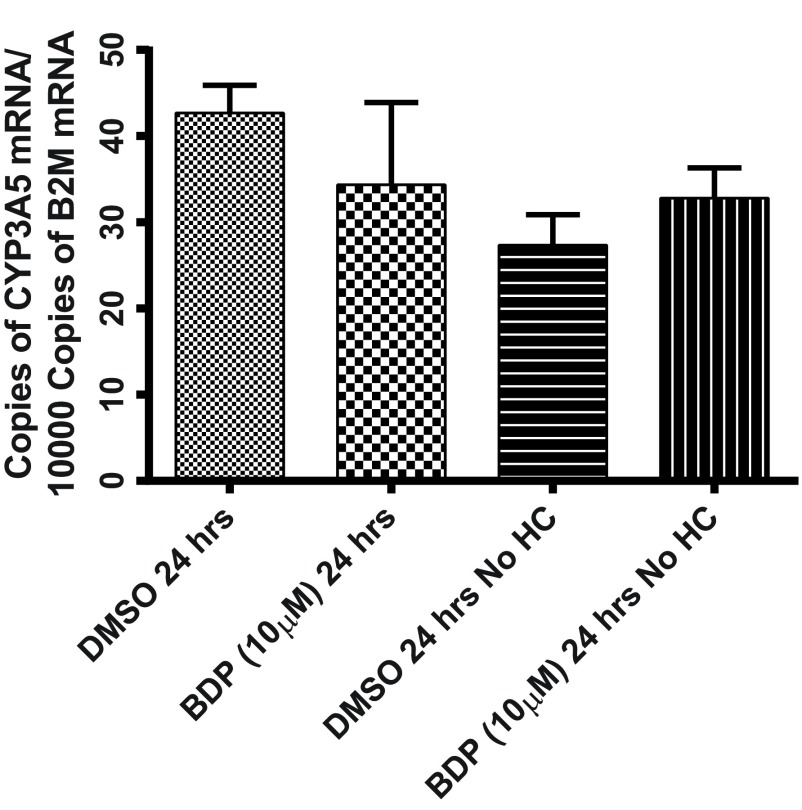
Beclomethasone dipropionate (BDP) treatment in SAEC cells results in no change in CYP3A5 mRNA. SAEC cells treated with BDP (10 µM; 24 h) or DMSO matching controls with and without hydrocortisone (HC). There was no significant difference between treatments or matching controls using one-way ANOVA with p<0.05.

SAEC cells from three separate donors were evaluated for CYP3A5*1 and other variant forms of CYP3A5 mRNA expression and induction in response to glucocorticoid treatment. Initial experiments demonstrated that mRNA for CYP3A5*1, but not CYP3A4 or 3A7, was expressed in one of the three SAEC samples (donor number 11662), but that expression levels were not altered by glucocorticoid treatment. It was hypothesized that the high concentration of hydrocortisone (500 µM) in the SAEC growth media prevented the induction of CYP3A5 mRNA by substantially lower concentrations of the glucocorticoids used in the treatments. Elimination of hydrocortisone from the media decreased the basal expression of CYP3A5 mRNA ( Figure 6). However, no change in mRNA abundance was observed over a 24 h treatment period with BDP. Furthermore, neither increasing the treatment concentration of BDP to 50 µM, nor treatment with [M1] at 150 µM led to an increase in CYP3A5 mRNA in SAEC cells. It was subsequently hypothesized that phthalates or other substances in the FBS might alter GR function and CYP3A5 mRNA induction by glucocorticoids ^27^. However, neither heat inactivation nor charcoal-stripping of the FBS in media with or without hydrocortisone led to CYP3A5 mRNA induction. The various manipulations to SAEC culture conditions and results for CYP3A5 induction are summarized in Table 3.

**Table 3.** Modifications made to SAEC culture media.

Basal culture conditions	Experimental modifications	Result
Cultured in growth media	-Heat inactivated media for treatment -10 µM BDP for 24 h	-Basal CYP3A5 mRNA expression observed in 1 out of 3 patients -No change in CYP3A5 mRNA
Cultured in growth media without hydrocortisone	-Heat inactivated media for treatment -10 µM BDP for 24 h	-Lowered basal level of CYP3A5 mRNA -No change in CYP3A5 mRNA
Cultured in growth media without hydrocortisone	-Heat inactivated media for treatment -50 µM BDP or 105 µM [M1] for 24 h	-No change in CYP3A5 mRNA
Cultured in growth media without hydrocortisone	-Treated in heat inactivated and charcoal stripped FBS -50 µM BDP or 105 µM [M1] for 24 h	-No change in CYP3A5 mRNA

Effect of inhaled glucocortcoids and their metabolites on CYP3A5 mRNA expression in human lung cells File 1: Data are peak areas for the metabolites beclomethasone-17-monopropionate ([M1], the active drug) and Δ6-beclomethasone dipropionate ([M5], the CYP3A-mediated metabolite) and the internal standard (IS, prednisolone). Treatment groups were beclomethasone dipropionate (BDP) alone, beclomethasone dipropionate (BDP) plus esterase inhibitors (EI), 1-aminobenzotriazole (1-ABT) plus beclomethasone dipropionate (BDP), and 1-aminobenzotriazole (1-ABT) plus esterase inhibitors (EI) plus the active drug [M1]. Data corresponds to Figure 3A in the main text.
File 2: Data are copy numbers of CYP3A5*1 mRNA and β-microglobulin (B2M, housekeeping gene) mRNA. Treatment groups are dimethyl sulfoxide (DMSO, vehicle control), beclomethasone dipropionate (BDP), beclomethasone dipropionate (BDP) plus esterase inhibitors (EI), 1-aminobenzotriazole (1-ABT), 1-aminobenzotriazole (1-ABT) plus beclomethasone dipropionate (BDP), and 1-aminobenzotriazole (1-ABT) plus esterase inhibitors (EI) plus active drug [M1]. Data corresponds to Figure 3B in the main text. 
File 3: Data are copy numbers of CYP3A5*1 mRNA and β-microglobulin (B2M, housekeeping gene) mRNA. Treatment groups were dimethyl sulfoxide (DMSO, vehicle control), beclomethasone dipropionate (BDP), ketoconazole (KTZ) at 50 µM plus beclomethasone dipropionate (BDP), ketoconazole (KTZ) at 10 µM plus beclomethasone dipropionate (BDP), and ketoconazole (KTZ) at 1 µM plus beclomethasone dipropionate (BDP). Data corresponds to Figure 4A in the main text. 
File 4: Data are copy numbers of CYP3A5*1 mRNA and β-microglobulin (B2M, housekeeping gene) mRNA. Treatment groups were dimethyl sulfoxide (DMSO, vehicle control), budesonide (BUD), ketoconazole (KTZ) at 50 µM plus budesonide (BUD), ketoconazole (KTZ) at 10 µM plus budesonide (BUD), and ketoconazole (KTZ) at 1 µM plus budesonide (BUD). Data corresponds to Figure 4B in the main text.
File 5: Data are copy numbers of CYP3A5*1 mRNA and β-microglobulin (B2M, housekeeping gene) mRNA. Treatment groups were dimethyl sulfoxide (DMSO, vehicle control), triamcinolone acetonide (TCL), ketoconazole (KTZ) at 50 µM plus triamcinolone acetonide (TCL), ketoconazole (KTZ) at 10 µM plus triamcinolone acetonide (TCL), and ketoconazole (KTZ) at 1 µM plus triamcinolone acetonide (TCL). Data corresponds to Figure 4C in the main text. 
File 6: Data are copy numbers of CYP3A5*1 mRNA and β-microglobulin (B2M, housekeeping gene) mRNA. Treatment groups were dimethyl sulfoxide (DMSO, vehicle control), fluticasone propionate (FLT), ketoconazole (KTZ) at 50 µM plus fluticasone propionate (FLT), ketoconazole (KTZ) at 10 µM plus fluticasone propionate (FLT), and ketoconazole (KTZ) at 1 µM plus fluticasone propionate (FLT). Data corresponds to Figure 4D in the main text.
File 7: Data are copy numbers of CYP3A5*1 mRNA and β-microglobulin (B2M, housekeeping gene) mRNA. Treatment groups were dimethyl sulfoxide (DMSO, vehicle control), flunisolide (FLN), ketoconazole (KTZ) at 50 µM plus flunisolide (FLN), ketoconazole (KTZ) at 10 µM plus flunisolide (FLN), and ketoconazole (KTZ) at 1 µM plus flunisolide (FLN). Data corresponds to Figure 4E in the main text. 
File 8: Data are copy numbers of CYP3A5*1 mRNA and β-microglobulin (B2M, housekeeping gene) mRNA. Control treatment groups were ketoconazole (KTZ) at 50 µM, ketoconazole (KTZ) at 10 µM, and ketoconazole (KTZ) at 1 µM in the main text.
File 9: Data are represented as copies of mRNA detected for the glucocorticoid receptor (GR) and β-microglobulin (B2M, housekeeping gene) for each treatment group. siRNA directed against the green fluorescent protein (GFP) was used as a negative control and compared to siRNA directed against the glucocorticoid receptor (GR) at 48, 72, and 96 hr post siRNA treatment. Data corresponds to Figure 5A in the main text. 
File 10: Data are copy numbers of mRNA detected for CYP3A5*1 and β-microglobulin (B2M, housekeeping gene). Pretreatments were siRNA directed against either green fluorescent protein (GFP, negative control) or the glucocorticoid receptor (GR). Three treatment groups consisted of dimethyl sulfoxide (DMSO, vehicle control), beclomethasone dipropionate (BDP), and beclomethasone dipropionate (BDP) plus esterase inhibitors (EI). Data corresponds to Figure 5B in the main text. 
File 11: Data are copies of CY3A5*1 mRNA divided by 10,000 copies of β-microglobulin (B2M, housekeeping gene) for each treatment. Treatments were dimethyl sulfoxide (DMSO, vehicle control), beclomethasone dipropionate (BDP), dimethyl sulfoxide (DMSO, vehicle control) treated cells cultured without hydrocortisone (HC), and beclomethasone dipropionate (BDP) treated cells cultured without hydrocortisone (HC). All treatments lasted for 24 hours. Data corresponds to Figure 6 in the main text.

## Discussion

Inhaled glucocorticoids are used to control undesirable symptoms in asthmatic patients. However, about 30% of the population does not benefit from this first-line treatment ^6^. Prior work demonstrated that the five most commonly prescribed glucocorticoids used in the treatment of asthma are metabolized by CYP3A enzymes, specifically CYP3A4, CYP3A5, and CYP3A7 ^10, 11^. Therefore, it has been proposed that unusually high rates of metabolism of glucocorticoids in lung cells by these enzymes might contribute to the decrease and/or lack of efficacy in some individuals. However, it is not understood how the expression of CYP3A enzymes is regulated in the lung in response to glucocorticoid treatment, despite extensive knowledge of this phenomenon in hepatocytes and the liver ^22^.

Using A549 cells, it was demonstrated that CYP3A5*1 mRNA was induced by glucocorticoid treatment ( Figure 3B and Figure 4A–E); neither CYP3A4 nor CYP3A7 mRNA were detected in A549 cells. Subsequent studies using a competitive antagonist of GR (ketoconazole) and siRNA selective for GR mRNA, demonstrated that inhibition of GR function prevented the induction of CYP3A5 mRNA by BDP and other glucocorticoids in A549 cells ( Figure 4A–E and Figure 5B). It was also demonstrated that CAR mRNA was not expressed by lung cells, consistent with previous data ^15^, and therefore could not be involved in the regulation of CYP3A5 expression by glucocorticoids as occurs in hepatocytes. It was concluded that CYP3A5 expression was directly regulated by GR ( Figure 2B). Schuetz *et al.* ^24^ previously described two “half sites” of GR (TGTTCT) separated by 160 bp in the promoter region of CYP3A5 in HepG2 cells and in human and rat hepatocytes. It was demonstrated that dexamethasone induced the expression of CYP3A5 by the GR homodimer binding to these two joined “half-sites” which could be blocked by RU-486, a GR antagonist. It is plausible these same sites are involved in the regulation of CYP3A5 in lung cells by BDP and other glucocorticoids.

Regardless of the exact mechanism of regulation, the current results illustrate that glucocorticoids have the capacity to induce the expression of CYP3A5 in A549 cells. These data, in conjunction with prior metabolism studies of glucocorticoids ^10, 11^, support the hypothesis that treating patients with glucocorticoids could increase levels of CYP3A5 in the lung, and therefore increase pulmonary glucocorticoid metabolism, ultimately increasing clearance, and potentially decreasing the concentration of active drug in lung cells. Though most of the population expresses the inactive form of CYP3A5 ( CYP3A5*3) ^13, 28^ those expressing CYP3A5*1, the active form of CYP3A5 ^13^, would exhibit increased clearance of the drug, and therefore could account for at least some of the 30% of patients who do not respond to inhaled glucocorticoid therapy.

In order to further support the hypothetical scenario above, the induction of CYP3A enzymes by glucocorticoids in various lung cells was studied. CYP3A5 mRNA expression was quantified in primary lung cells, which presumably more closely model epithelial cells of the human respiratory tract and lung. NHBE, lobar, and cells recovered from tracheal washes of mechanically ventilated children were evaluated for CYP3A enzyme expression and induction by glucocorticoids. Results in Table 2 show that CYP3A mRNA was not expressed in cells of the conducting airways in response to glucocorticoid treatment, indicating that these epithelial cells likely do not play a role in CYP3A-dependent metabolism of glucocorticoids in the lung. In contrast, select donor samples of SAEC cells, representing cells of the distal bronchioles, alveolar ducts, and alveoli, did express CYP3A5 ( Table 2). However, there was no change in CYP3A5 mRNA when these cells were treated with glucocorticoids. A thorough examination of potential confounding issues associated with cell culture revealed a high concentration of hydrocortisone (500 µM) in the growth media. Because cells were treated with only 10 µM BDP, it would stand to reason that no change in CYP3A5 mRNA would occur because CYP3A5 expression would already be maximized as a result of hydrocortisone activating the GR pathway.

Experiments conducted in A549 cells showed that culturing cells in 500 µM hydrocortisone increased the basal expression of CYP3A5 mRNA by 2-fold, masking the induction routinely observed using 10 µM BDP for 24 h. When A549 cells were subsequently cultured in media without hydrocortisone for 48 h, providing sufficient time for a “wash out” of the hydrocortisone, the basal expression of CYP3A5 mRNA was reduced, and ~2-fold induction of CYP3A5 mRNA occurred with the 10 µM BDP, 24 h treatment. Therefore, hydrocortisone was omitted from the SAEC growth media. Subsequent experiments in SAEC cells showed no change in CYP3A5 mRNA in response to glucocorticoid treatment ( Figure 6), albeit removal of hydrocortisone from the media caused a slight decrease in the basal level of CYP3A5 mRNA expression, suggesting that GR plays a role in the regulation of CYP3A5. It is feasible that because cells had been exposed to such high concentrations of hydrocortisone during their isolation and expansion, that 10 µM of BDP was not sufficient to induce CYP3A5 mRNA, even after culturing the cells in the absence of hydrocortisone for multiple division cycles. Therefore, the concentration of BDP was increased to 50 µM and an additional treatment group using 150 µM [M1] was added. Again no increases in CYP3A5 mRNA was observed. Heat-inactivated and charcoal-stripped FBS were also utilized to remove potential interfering compounds from FBS, and still no change was observed.

To our knowledge, no one has observed a change in CYP3A mRNA expression in any primary human lung cell cultures. However, Cyp3a11, 3a13, and 3a16 mRNA and protein induction have been documented in mouse lung following dexamethasone treatment ^29^. As such, additional studies using animal models and relevant samples from human patients need to be evaluated in order to conclusively confirm or reject the hypothesis that CYP3A genes are regulated in human subjects, particularly asthmatics, in response to glucocorticoid treatment since current *in vitro* models remain unexplainably limited in value for such studies.

In summary, the data presented herein demonstrate that, in A549 cells, glucocorticoid binding to the glucocorticoid receptor regulates the expression of CYP3A5. However, further research is needed to determine if changes in CYP3A5 expression occur in the human respiratory tissue similar to A549 cells, the precise mechanism by which this process occurs, and whether changes in the local metabolism of glucocorticoids by CYP3A5 ultimately impact glucocorticoid efficiency in asthma patients refractory to glucocorticoid treatment. Because we have not been able to evaluate this mechanism more thoroughly in primary lung cells, particularly from asthmatic subjects, the physiological and/or clinical relevance of the present study in steroid insensitive patients requires further investigation.
