# Supplementary material for: Regulation of CYP3A genes by glucocorticoids in human lung cells
Source: F1000Res. 2013 Oct 8;2:173. Originally published 2013 Aug 13. [Version 2] doi: 10.12688/f1000research.2-173.v2 (PMC3869485; doi:10.12688/f1000research.2-173.v2)
Supplement: Peer review history

# Peer review: Referee response for version 2

**Published:** 2013-11-14

**Authors:** Philip Ballard

**Affiliations:** 1 University of California San Francisco, San Francisco CA, USA

The revisions are appropriate and the paper is improved.

I have read this submission. I believe that I have an appropriate level of expertise to confirm that it is of an acceptable scientific standard.

# Peer review: Referee response for version 2

**Published:** 2013-10-25

**Authors:** Yassine Amrani

**Affiliations:** 1 Department of Infection, Immunity and Inflammation, University of Leicester, Leicester, UK

I have no further comments for the authors. All the previous concerns from my review have been adequately addressed.

I have read this submission. I believe that I have an appropriate level of expertise to confirm that it is of an acceptable scientific standard.

# Peer review: Referee response for version 1

**Published:** 2013-09-18

**Authors:** Yassine Amrani

**Affiliations:** 1 Department of Infection, Immunity and Inflammation, University of Leicester, Leicester, UK

This article explores for the first time the expression of cytochrome P450 enzymes (CYP3A5) in lung cells. The merits of the present study are the investigation of CYP3A5 expression in different primary cell lines and the use of various corticosteroids in well-controlled experiments that have produced solid data.

 

Main concern:

Although the data in A549 cells are convincing, the hypothesis that CYP3A5 is expressed in “non-transformed” lung cells (tracheal/bronchial) potentially regulated by corticosteroid therapy is not apparent from the study. Unless the authors can show that CYP3A5 is indeed expressed in vivo in the epithelium in endobronchial biopsies from asthmatic patients, the physiological and/or clinical relevance of the present study in steroid insensitive patients is not apparent and this point should be revised in the discussion.  

Another concern relates to the data shown in figure 3A. The figure shows that the CYP-P450 suicide inhibitor (1-ABT) blocks the production of BDP active metabolite M1.This is a surprising finding as production of M1 is thought to result from the action of esterases in contrast to the production of inactive compounds (M5) occurs via CYP-P450 enzyme. How can the authors explain that M1 is sensitive to 1-ABT?

I have read this submission. I believe that I have an appropriate level of expertise to confirm that it is of an acceptable scientific standard.

# Peer review: Referee response for version 1

**Published:** 2013-08-21

**Authors:** Philip Ballard

**Affiliations:** 1 University of California San Francisco, San Francisco CA, USA

This paper addresses mechanisms of glucocorticoid metabolism in various cell lines and primary lung cells in vitro. In general, the experimental approaches are appropriate and the results are clearly presented.  

The following minor changes are suggested to improve clarity and data interpretation:

1. In paragraph 1 of page 4, state explicitly whether hydrocortisone was present in the culture medium of the other cell lines and primary cultures.

1. In paragraph 3 of page 8 and the final paragraph page 9; I suggest caution in the conclusion that the results, which were obtained in an adenocarcinoma cell line, support the proposal that glucocorticoids increase CYP3A5 and inhaled glucocorticoid metabolism in vivo. In fact, the negative data for primary cells argues against the hypothesis. 

I have read this submission. I believe that I have an appropriate level of expertise to confirm that it is of an acceptable scientific standard.
